# Associated Factors of Suicidal Thoughts in HIV-Positive Individuals

**Published:** 2015-06

**Authors:** Fatemeh Dabaghzadeh, Fatemeh Jabbari, Hossein Khalili, Ladan Abbasian

**Affiliations:** 1 Department of Clinical Pharmacy, Faculty of Pharmacy, Kerman University of Medical Sciences, Kerman, Iran; 2 Department of Clinical Pharmacy, Faculty of Pharmacy, Tehran University of Medical Sciences, Tehran, Iran; 3 Iranian Research Center for HIV/AIDS, Tehran University of Medical Sciences, Tehran, Iran

**Keywords:** *Suicidal ideation*, *HIV- Positive Individuals*

## Abstract

**Objective:** As a first study, suicidal ideation and its correlates have been evaluated in Iranian HIV positive population.

**Methods:** One hundred and fifty HIV-positive individuals were recruited in this cross-sectional study. The Hospital Anxiety and Depression Scale (HADS), Positive and Negative Suicide Ideation (PANSI), Pittsburgh Sleep Quality Inventory (PSQI) and Somatization subscale of Symptom Checklist 90 (SCL 90) as self- reported questionnaires were used to assess the patients’ anxiety and depression status, suicidal thoughts, sleep quality and physiological factors, respectively.

**Results:** Antiretroviral therapy and efavirenz intake did not show any significant effects on the patients’ suicidal ideation. Anxiety (p<0.001), depression (p<0.001), poor physical activity (P<0.001) and sleep quality (p<0.001) were significantly associated with the patients’ negative suicidal ideation. From the patients’ demographic data, unemployment (p = 0.04), living alone (p = 0.01), and lack of family support (p = 0.01) were correlated with the patients’ negative suicidal thoughts.

**Conclusion:** Although hospitals are the main referral centers for providing care for HIV-positive individuals in Tehran, Iran, conducting a multi-center study with sufficient sample size from different areas of our country that include individuals with different behaviors and cultures is essential to confirm the results of this study.

Suicide is a worldwide public health problem. It is one of the major leading cause of death in different populations (1). The suicidality is categorized into suicidal ideation, suicide attempts and completed suicide (2). Suicidal ideation is defined as any self-reported thoughts of engaging in suicide-related behavior (3). Suicidal ideation is more common than suicide attempt and completed suicide, and the presence of suicidal ideation increases the risk of suicidal attempt and completed suicide (4, 5). 

Psychiatric disorders are common complaints in patients with chronic diseases including Human Immunodeficiency Virus (HIV) positive individuals (6-9)]. HIV infection itself can affect the patients’ quality of life and cause mental problems (10). It has been shown that suicidal ideation is more common in HIV positive patients in comparison with the general population (6, 11, 12). It has been reported that suicide attempt rate in individuals with AIDS is 7.4 times higher than the general population (13). The correlation between suicidal ideation and HIV infection is not well described, but it can be explained by the bio-psychological aspects of HIV (5). Following the introduction of effective antiretroviral therapy (ART), suicidal behavior is declined in HIV- positive people (6), but its rate may be increased during the first month of starting ART regimens which contain efavirenz due to the psychiatric adverse effects of efavirenz (14).

Several associated factors have been reported for suicidal ideation in HIV positive individuals. These factors increased the risk of suicide (6, 11). These factors consisted of mental disorders (such as depression and anxiety), feelings (such as hopelessness), severity of the disease, HIV related physical symptoms, antiretroviral regimen, CD4 count, ART related adverse drug reactions, socioeconomic factors (such as living single, low income, social support, discrimination and stigmatization) and patients’ demographic factors (such as advanced age, female sex, substance and alcohol abuse) (4, 6, 15). On the other hand, protective factors such as positive social support and social cohesion may decrease the risk of suicide ideation (5). It is essential to detect the protective and negative risk factors of suicidal ideation to prevent suicide (5).

The rates of suicide in Asian countries such as Iran are moderate (16). The prevalence of suicidal ideation was reported to be 10 to 12.7% in the general population of Iran (1, 16). Internal stigma and discrimination are higher in Iran in comparison with other countries. The reasons for these differences are due to the culture and acceptance of the Iranians. Internal stigma and discrimination are related to suicidal ideation in HIV- positive individuals (17). As a first study in Iranian HIV- positive population, the goal of this study was to examine the associated factors of suicidal ideation in HIV- positive individuals.

## Material and Methods

This cross-sectional study was conducted in the HIV Clinic of Imam Khomeini Hospital, Tehran, Iran during May 2013 to May 2014. The study protocol was approved by the hospital’s Ethical Committee and all participants signed the informed contest form. One hundred and fifty HIV- positive individuals whose infections were confirmed based on the enzyme-linked immunosorbent assay (ELISA) and Western-Blot tests were recruited in this study. These participants consisted of HIV- positive people who did not receive ART (n = 50), patients who received ART including efavirenz (n = 78), and patients who received ART without efavirenz (n = 22). Patients’ demographic data including age, sex, weight, education, employment, marital and living status, housing, family support condition, substance and alcohol abuse, time from HIV infection diagnosis, duration of ART, concomitant diseases and medications and route of HIV transmission were collected from their medical record. The validated Persian versions of self- reported questionnaires were used to assess the psychological and physiological factors. The Hospital Anxiety and Depression Scale (HADS) consists of 14 items and measures two constructs of anxiety (7 items) and depression (7 items); each item is rated from 0 to 3 (18, 19) with cut-off points for severity (scores: 0–7 normal; 8–10 mild; 11–14 moderate; and 15–21 severe). Positive and Negative Suicide Ideation (PANSI) scale includes 14 items, which is scored from 0 to 4 for each item, based on symptoms severity. PANSI scale has two subscales of questions: 6 items for positive suicidal ideation and 8 items for negative suicidal ideation (14, 20, 21). PANSI scale evaluates both positive and negative thoughts over past two weeks. The risk of suicidal behaviors increases with more negative thoughts and less positive thoughts (20). Most existing self-report questionnaires of suicide ideation do not asses protective factors (21). PANSI can be used as simple risk-screening tool and predict future suicide attempts. Decreased positive ideation and increased negative suicidal ideation contribute to subsequent suicide attempts (22). It meant HIV positive individuals who had high negative suicidal thought may still report some desire or wish to live (21). Pittsburgh Sleep Quality Inventory (PSQI) was used to evaluate the participants’ sleep quality during the study period. PSQI is a 7-component questionnaire (subjective sleep quality, sleep latency, sleep duration, habitual sleep efficiency, sleep disturbances, use of sleeping medications, and daytime dysfunction). Patients with a total score of greater than 5 are known as poor sleepers (23, 24). Somatization subscale of Symptom Checklist 90 (SCL 90) was used to assess the patients’ physiological factors. Somatization dimension of SCL-90 represents physical morbidity and includes 12 items. This subscale represents distress in different systems such as cardiovascular, gastrointestinal, respiratory, autonomic systems and is rated from 0 (none) to 4 (extreme) for each item (25-27).

The statistical package of social science (SPSS) version 16 was used for all analyses. Normal distribution of the data was evaluated by Kolmograph Smirnov test. Spearman rank test was used to determine the correlation between PANSI scores and other quantitative variables. Mann–Whitney-U-test was used to compare the quantitative data between the two groups. Kruskal–Wallis one-way analysis of variance was used to compare more than two quantitative variables. The Dunn test was performed as a post-hoc test in the analysis of variance. Chi-square test was used to compare the demographic data between the HIV positive patients and the control group. P values of less than 0.05 were considered as statistically significant.

## Results

The included HIV- positive individuals consisted of 100 (67 %) males and 50 (33 %) females. The mean ± SD age of these participants was 35.92 ± 7.75 years. Demographic data of HIV-positive individuals were summarized in Table 1. There was not statistically significant difference in positive (p = 0.413) and negative (p = 0.133) suicidal ideation scores among HIV- positive patients who received ART, ART including efavirenz, and ART without efavirenz. Receiving efavirenz did not show any significant effect on the positive (p = 0.18) and negative (p = 0.07) suicidal ideation scores in the HIV positive individuals. Data regarding the measured scales of the study are presented in Table 2. Statistically significant correlation was found between the negative suicidal ideation and PSQI (r = 0.44, p<0.001), somatization subscale of SCL-90 (r = 0.45, p<0.001), depression (r = 0.54, p<0.001), and anxiety (r = 0.54, p<0.001) scores in the HIV positive people. On the other hand, a statistically significant indirect correlation was found between positive suicidal ideation and PSQI (r = 0.37, p< 0.001), somatization subscale of SCL-90 (r = 0.36, p< 0.001), depression (r = 0.60, p< 0.001), and anxiety (r = 0.58, p<0.001) scores in this population. 

**Table 1:** Summery of baseline demographic characteristics of the patients

**Variable **		**Number (%)**
Sex	Male	100 (67 %)
	Female	50 (33 %)
Education	Primary school	16 (10. 67 %)
	Guidance school	62 (41.33 %)
	High school	52 (34.67 %)
	Advanced	20 (13.33 %)
Employment	Employed	86 (57.33 %)
	Unemployed	64 (42.67 %)
Marital status	Married	75 (50 %)
	Single	61(40.67 %)
	Divorced	14 (9.33 %)
Living alone	With family	130 (86.67 %)
	alone	20 (13.33 %)
Housing	Have house	137 (91.33 %)
	Do not have house	13 (8.67 %)
Family support	Yes	130 (86.67 %)
	No	20 (13.33 %)
Smoking	Yes	87 (58 %)
	No	63 (42 %)
Substance abuse	Yes	80 (53.33 %)
	No	70 (46.67 %)
Alcohol use	Yes	19 (12.67 %)
	No	131 (87.33 %)
Route of transmission	Sexual contact	76 (50.67 %)
	Injection drug Use	64 (42.67 %)
	Blood	8 (5.33 %)
	Unknown	2 (1.33 %)

**Table 2:** Anxiety, depression, somatization, suicidal ideation and sleep quality status of the patients

**Group**	**HAS** 1 **Median** ** (interquartile range** **25-75)**	**HDS** 2 **Median** ** (interquartile range** **25-75)**	**Somatization subscale of SCL 90** 3 **Median** ** (interquartile range** **25-75)**	**PSI ** 4 **Median** ** (interquartile range** **25-75)**	**NSI ** 5 **Median** ** (interquartile range** **25-75)**	**PSQI ** 6 **Median** ** (interquartile range** **25-75)**
All HIV-positive patients as a group	8.50 (5-13)	7.00 (4-11)	11.00 (5-19)	21.00 (15-24)	10.00 (8-17)	6.00 (4-11)
HIV- positive individuals who did not receive antiretroviral	10.50 (5.75-14.25)	8.50 (5-13)	14.00 (6.5-20)	19.00 (14.75-24.25)	12.00 (8.75-22.5)	6.00 (4-12)
People who received antiretroviral including efavirenz	6.00 (3.75-10)	9.00 (8-16)	21.00 (16.75-24.25)	9.00 (5-17.5)	7.00 (5-11.25)	7.00 (4-11)
People who received antiretroviral without efavirenz	7.00 (4-12)	9.500 (5-12.25)	9.50 (3.75-23.25)	20.00 (14.75-24.5)	10.00 (8-16.25)	9.00 (4-11)

1 Hospital anxiety scale

2 Hospital depression scale

3 Symptom check list

4 Positive suicidal ideation

5 Negetive suicidal ideation

6 Pittsburgh Sleep Quality Inventory

**Table 3:** Associated factors of positive and negative suicidal ideations in the patients

**Variable **	**P value ** 1	
	**NSI ** 2	**PSI ** 3
Sex	0.58	0.12
Education	0.39	0.35
Employment	0.04	0.06
Marital status	0.71	0.08
Living alone	0.01	0.18
Housing	0.13	0.71
Family support	0.01	0.22
Smoking	0.24	0.91
Substance abuse	0.41	0.06
Alcohol use	0.06	0.21
Route of transmission	0.11	0.03
age	0.31	0.09
Weight	0.61	0.58
Time from HIV infection diagnosis	0.32	0.13
duration of antiretroviral therapy	0.39	0.15
Receiving antiretroviral therapy	0.06	0.33
Receiving efavirenz	0.07	0.18
Hospital anxiety scale	<0.001	<0.001
Hospital depression scale	<0.001	<0.001
Somatization subscale of SCL 90^4^	<0.001	<0.001
^ 7^Pittsburgh Sleep Quality Inventory	<0.001	<0.001

1 Based on Kruskal–Wallis or Mann–Whitney-U-test or Spearman rank test analysis

2 Negetive suicidal ideation

3 Positive suicidal ideation

4 Symptom checklist

No a statistically significant correlation was detected between positive and negative suicidal ideation scores and patients’ age, weight [70 (63-77) kg], time from HIV infection diagnosis [27 (8-72) months] and duration of ART [24 (11-49) months]. Unemployment (p = 0.04), living alone (p = 0.01), and lack of family support (p = 0.01) showed significant negative effects on the HIV positive individuals’ negative suicidal ideation scores. From the demographic data, only the route of HIV infection transmission (p = 0.01) had a significant effect on the patients’ positive suicidal ideation scores. People acquiring HIV infection from sexual contact had the lowest positive suicidal ideation scores. The patients’ concomitant diseases or medications did not show any significant effect on their positive and negative suicidal ideation scores. Probable associated factors of positive and negative suicidal ideation in the patients were summarized in Table 3. Based on the PSQI score, 83 (55.3%) of the HIV positive people were categorized as persons with sleep problems. Mild, moderate and severe anxiety symptoms were detected in 27 (18%), 31 (20.67%) and 25 (16.67%) of the HIV positive patients, respectively. The patients’ depression scores were categorized as mild, moderate and severe in 29 (19.33%), 29 (19.33%) and 16 (10.66%) of the total studied patients, respectively. The anxiety and depression scores of 67 (44.67%) and 76 (50.67%) of the patients were within the normal range, respectively.

## Discussion

Compatible with other studies (6, 28, 29), this study revealed that negative suicidal thoughts are prevalent in Iranian HIV positive individuals receiving efavirenz and that other ART medications did not have any significant effects on suicidal ideation in Iranian HIV- positive individuals. However, one previous study has reported that efavirenz can increase the suicidal thought during the first month of ART (14). The duration of efavirenz containing or not containing ART for the majority of our participants was more than one month. Anxiety, depression, sleep quality, physical morbidity, employment, living single, family support and sexual contact (as route of HIV infection transmission) were detected as the suicidal ideation correlates. Some of these findings were consistent and some were in contrast to the findings of other surveys. Keiser et al. reported the results of the Swiss HIV Cohort Study in 15,275 HIV-positive patients. Their self-designed questionnaire on the mental disorders was used in that cohort. They reported higher rate of suicide in HIV positive patients compared with the general Swiss population. They found that suicide rates declined with the introduction of ART and increasing CD4 cell counts. Older age, male sex, Swiss nationality, advanced clinical disease, positive history of injection drug use and antipsychotic treatment were significant associating factors that increased suicide rates (6). In a cross- sectional study by Govender et al. on 156 newly diagnosed HIV-positive patients in Durban, South Africa, the Beck Hopelessness and Beck Depression Inventory (BDI) scales were used to evaluate suicidal ideation and depression in the included individuals. A direct relationship was detected between the people’s depression scores and suicidal ideation in this study (30). Also, Lawrence et al. examined the associated conditions of suicidal ideation, using computerized patient-reported outcome assessments in 1216 patients living with HIV infection in the United States. They used 9-item Patient Health Questionnaire (PHQ-9) to assess the suicidal ideation. Older age, active substance abuse and severe depression increased the frequency of suicidal ideation in this cohort (4). In another study by Carrico et al., conducted on 2909 HIV- positive patients in the United States, regular marijuana use, severe HIV symptoms, ART side effects and more depressive symptoms were detected as significant associated factors with the patients’ suicidal thoughts. On the other hand, Hispanic/Latino race, social support and greater coping self-efficacy declined suicidal ideation in this population (11). The results of Guimaraes et al. cross- sectional study showed that non-white skin color, unemployment, and diagnosis of major depression could be considered as risk factors for suicide in patients with chronic infectious diseases (including HIV infection) in Brazilian population (12). Unemployment, living alone, suicidal ideation and having more than 2 psychiatric/cognitive risk factors increased the risk of suicide and accidental or violent death in a case control study by Mcmanus et al. in the HIV-positive Australian population. However, CD4 cell count of >500 cells/µl decreased this risk in men living with HIV infection (31). In another study by Shittu et al., a significant correlation was found between hopelessness, depression and suicidal ideation. This study was performed among HIV- positive depressed patients in Nigeria, West Africa, using PHQ-9 for suicidal ideation assessment (5). Jia et al. investigated the risk factors for subsequent suicide in HIV positive patients in Denmark. They found that the patients’ low income, living alone, dwellers of the Capital area, and having other psychiatric illnesses can increase the risk of subsequent suicide attempts (32). Predictors of suicidal ideation in HIV-positive women in the United States using Harkavy Asnis Suicide Survey reported AIDS diagnosis, psychiatric symptoms, and physical or sexual abuse as negative predictors, and having children and being employed as positive predictors of both suicidal ideation and attempts (2). Haller et al. identified that depression was the most common psychiatric disease diagnosis among HIV- positive people with suicidal ideation. They did not find any correlation between suicidal ideation and the patients’ demographics data including sex, route of HIV infection transmission, housing, employment, marital status, education and living status. They developed an interview survey with seven items to evaluate suicidal ideation (33). In our study, when the route of HIV transmission was sexual contact, people had less positive suicidal ideation. It may be due to the religion and culture of the Iranians that caused those who had illegal sexual relations to feel guilty. We could not find any association between age, sex, marital status, education, housing condition, or substance and alcohol abuse and suicidal ideation. Different scales and questionnaires were used to assess suicidal ideation in HIV-positive individuals in the current studies that can be one important source of different results. On the other hand, culture, religion and nationality can affect mental disorders such as suicidal ideation in HIV- positive population (6, 17, 34, 35).

## Limitations

 The main limitations of this study are the small sample size, being conducted in a single center, and heterogeneity of the included patients. Data from different parts of the country are needed for a national conclusion. 

## Conclusion

This was the first study that has evaluated the associated factors of suicidal ideation in Iranian HIV- positive individuals. Valid and Reliable Persian versions of HAS, HDS, SCL-90, PSI, NSI, PSQI scales were used to assess the patients’ anxiety, depression and somatization scores, positive and negative suicidal thoughts and sleep quality, respectively. Treatment with ART and efavirenz intake did not show any significant effects on the patients’ suicidal ideation. Anxiety, depression, poor physical morbidity and sleep quality were significantly associated with the patients’ negative suicidal ideation. From the patients’ demographic data, unemployment, living alone, and lack of family support were correlated with the patients’ negative suicidal thoughts. 
